# Immune Priming with Spatially Fractionated Radiation Therapy

**DOI:** 10.1007/s11912-023-01473-7

**Published:** 2023-11-18

**Authors:** Lauren Lukas, Hualin Zhang, Karen Cheng, Alan Epstein

**Affiliations:** 1 Department of Radiation Oncology, Keck School of Medicine, University of Southern California, Los Angeles, CA USA; 2 Department of Pathology, Keck School of Medicine, University of Southern California, Los Angeles, CA USA

**Keywords:** Spatially fractionated radiation therapy, Immunomodulation, Radioimmunotherapy, Immunotherapy

## Abstract

**Purpose of Review:**

This review aims to summarize the current preclinical and clinical evidence of nontargeted immune effects of spatially fractionated radiation therapy (SFRT). We then highlight strategies to augment the immunomodulatory potential of SFRT in combination with immunotherapy (IT).

**Recent Findings:**

The response of cancer to IT is limited by primary and acquired immune resistance, and strategies are needed to prime the immune system to increase the efficacy of IT. Radiation therapy can induce immunologic effects and can potentially be used to synergize the effects of IT, although the optimal combination of radiation and IT is largely unknown. SFRT is a novel radiation technique that limits ablative doses to tumor subvolumes, and this highly heterogeneous dose deposition may increase the immune-rich infiltrate within the targeted tumor with enhanced antigen presentation and activated T cells in nonirradiated tumors.

**Summary:**

The understanding of nontargeted effects of SFRT can contribute to future translational strategies to combine SFRT and IT. Integration of SFRT and IT is an innovative approach to address immune resistance to IT with the overall goal of improving the therapeutic ratio of radiation therapy and increasing the efficacy of IT.

## Introduction

Immuno-oncology drugs, in particular immune checkpoint inhibitors (ICIs), have revolutionized the treatment landscape of several cancer types [1]. Although there may be high levels of programmed death ligand 1 (PD-L1) expressed on both tumor and immune cells, monotherapy with anti-PD-1/PD-L1 antibodies may have limited antitumor activity and a small clinical benefit, and development of immune-oncology strategies has moved toward a combination approach with ICIs to overcome primary and acquired immune resistance [1]. Although these combination approaches usually consist of systemic therapies, recent studies have shown that radiotherapy (RT) can modify the tumor microenvironment (TME) to induce a systemic antitumor immune response through proinflammatory cytokines and engagement of the innate and adaptive immune systems leading to abscopal effect and response to immune checkpoint blockade (ICB) in distant nonirradiated sites [2]. Although an abscopal effect has been reported, the overall occurrence rate is low [2]. Both conventional fractionation and ablative radiation regimens have limitations in terms of immune effects, since they homogeneously target the tumor with an additional margin, and thus, the majority of circulating naïve T cells at critical points of cross-presentation are depleted and there is also toxicity to surrounding normal organs [3]. Primary immune resistance to IT and lack of substantial abscopal responses with combination systemic therapies or with ablative RT are significant and critical problems.

Novel radiation modalities, such as spatially fractionated radiotherapy (SFRT), have shown promise of increasing the therapeutic index of radiation therapy with the potential of immunomodulation [3]. SFRT consists of delivering a high ablative dose to small partial volume(s) within a tumor along with constraining the peripheral doses to the surrounding normal tissue [4]. This peak and valley distribution of SFRT might induce unique systemic effects due to the varying damage induced by dose or spatial placement of the beams, and it has been suggested that different dose and fractionation schedules may be a route to more consistent generation of abscopal responses [5••, 6, 7]. These findings suggest that SFRT as a component of combination radioimmunotherapy may create interspersed areas of intratumoral immune cell sparing and vascular access with the potential for better immune system activation [5••]. Release of interleukins and cytokines or other humoral mediators (e.g., IL-6, IL-8, TGFβ, TNFα, and reactive oxygen and nitrogen species) play a role in these pathways [8]. Early infiltrating lymphocytes have been shown to migrate from the surrounding tissue and the irradiated volume, whereas lymphocytes from lymph nodes maintain long-term antitumor activity [9]. PD-L1 has also been shown to be upregulated in abscopal tumors from SFRT-treated mice and the intratumoral immune cell composition of abscopal tumors showed significantly increased amounts of both activated CD4+ and CD8+ T cells [5••]. The findings illustrate the beginnings of what appears to be a mounting antitumor immune effect that could be instrumental to developing long-term abscopal tumor control in combination with IT [5••]. In this review, we examine the nontargeted immune effects of SFRT, summarize the evidence in preclinical and clinical studies, and discuss translational strategies to increase the efficacy of IT using SFRT immune priming.

## Radiation and the Immune System

The immune response to radiation is regulated by complex interactions among a variety of immune components and soluble mediators [10]. Following radiation, cells release fragments of cytosolic DNA, mitochondrial DNA, calreticulin, ATP, and other components, comprising damage-associated molecular patterns (DAMPs), which result in molecular cascades that activate the innate immune responses and immunogenic cell death [11]. Secretion of inflammatory mediators and cytokines is an initial, nonspecific acute reaction, or “cytokine storm,” that usually resolves within 24 h [10, 12]. The innate immune response consists of monocytes, which differentiate into dendritic cells (DCs) and inflammatory macrophages [10]. The function of macrophages includes phagocytosis of apoptotic bodies, presentation of antigens, cytotoxic activity, and secretion of cytokines, reactive oxygen species (ROS), and nitrogen oxide (NO) [10, 13]. The adaptive immune system consists of T cells, which are responsible for cell-mediated immune responses, and B cells, which are part of the humoral immune response mediated by antibodies. The adaptive immune response is involved in the development of immunological memory [10, 14]. Following radiation, antitumor immune responses are triggered by the release of tumor antigens and proinflammatory factors that can promote DC maturation and T cell activation [10]. Other mechanisms by which radiation affects immune responsiveness include alterations in the secreted cytokine profile, expression of calreticulin, and release of alarmins and nuclear proteins, including high mobility group box 1 protein (HMGB1) which serves as an endogenous ligand for toll-like receptor (TLR-4) [10, 15]. The induction of antitumor immune response, overcoming tumor radioresistance, and the ability to generate antitumor nontargeted effects can contribute to treatment success [10].

The concept that tumor sterilization requires comprehensive radiation of the entire tumor to high doses has long been a tenet of radiation oncology [3]. In order to achieve this goal of delivering a high dose while limiting toxicity to surrounding normal organs, radiation has typically been delivered with conventional fractionation or extremely hypofractionated (ablative) regimens. Conventional fractionation regimens deliver small doses of 1.8–2 Gy per day over weeks of treatment with total doses of 60–80 Gy, and ablative regimens, such as stereotactic radiosurgery (SRS) or stereotactic ablative radiotherapy (SABR), deliver large single fractions of up to 25–34 Gy or up to 60 Gy in 3–8 fractions over 1–2 weeks [3].

Conventional fractionation takes advantage of radiobiological effects, known as the “4 R’s of radiobiology,” which consist of repair of DNA damage, redistribution of cells in the cell cycle, repopulation, and reoxygenation of hypoxic tumor area [16]. The vast majority of experimental studies that were used to originally define the 4 R’s were based on clonogen survival in hierarchical normal tissues or tumors, such as in vitro clonogenic cell survival assays, in vivo splenic colony-forming unit or colonic/jejunal crypt cell assays, or tumor regrowth assays and evaluated responses in the short term [16]. Normal and also malignant stem cells, for example, in the normal colon, in bone marrow (long-term repopulating hematopoietic stem cells), or in melanoma, cycle very slowly and would not be evaluated by most of the standard radiobiological assays [16]. Complete cell kill is required, however, to prevent a recurrence and this is determined by a composite of the radioresistance of different subpopulations and the number of cells with that level of radioresistance [16]. Radioresistance also occurs with conventionally fractionated doses of 1.8–3 Gy, as the resulting reactive oxygen species (ROS) induce translation of preformed hypoxia-inducible factor (HIF-1) mRNA transcripts. Ensuing upregulation of proangiogenic elements was observed, including vascular endothelial growth factor (VEGF), which interrupts normal apoptotic pathways and works to facilitate radioresistance [17].

Ablative regimens are possible due to physical separation of dose from normal tissues using limited target volumes and highly conformal dose delivery [3]. Ablative regimens, however, can have both immunogenic and immunosuppressive effects on the tumor microenvironment (TME). Cancer cells survive the host immune system by creating immunosuppressive TMEs including immune cells such as regulatory T cells, MDSCs and tumor-associated macrophages (TAMs) that impair infiltration of CD8^+^ T cells that mediate antitumor responses [3]. T cells and B cells are radiation sensitive while regulatory T cells, macrophages, DCs, and NK cells are radiation resistant [3]. It has been shown that ablative or hypofractionated regimens induce superior CD8^+^ T cell antitumor responses compared to conventional fractionation regimens, while the addition of fractionated radiation may result in death of infiltrating T cells that kill tumor cells [3]. Ablative doses of radiation can cause vascular injury, especially above 10 Gy, which has been shown to induce hypoxia, acidification of the TME, and indirect death of tumor cells [18]. SABR has been shown to increase vascular permeability and apoptosis through the ceramide pathway [19]. Vascular endothelial injury exacerbates platelet aggregation and thrombosis formation, which then further blocks the blood vessels. High-dose radiation induced blood vessel injury and ischemia further leads to tumor necrosis. Consequently, the antitumor effect of radiotherapy is enhanced [18]. However, the long-term vascular damage that leads to chronic inflammation and subsequent vascular occlusion is also responsible for damage to healthy tissues responsible for chronic complications [19].

Prospective data advocate the use of SBRT in a broad clinical context including the management of metastatic lesions or in combination with immune checkpoint blockade to optimize local responses, but while these studies demonstrate efficacy in limiting disease progression, a clear method to also affect and potentially eradicate metastases has yet to emerge with SBRT [5••]. Piper et al. [20] showed that a combination of radiation and PD1-IL2v immunotherapy enhances CD8^+^ T cell polyfunctionality, activation, and immune memory across tumor, lymph node, and blood compartments and results in a durable local and systemic antitumor response. In their murine pancreatic cancer model, a single 8-Gy dose of X-ray radiation was delivered to mouse pancreata at 7 days post-implantation. Tumor-specific CD8^+^ T cells were shown to more frequently infiltrate the TME; highly express a myriad of activation markers, including IFNg, CD44, CD69, and CXCR3; and were more efficient at directly killing cancer cells when treated with radiation + PD1-IL2v. In the absence of radiation, the response to PD1-IL2v treatment was significantly reduced [20]. Demaria et al. [21] reported that “hot” tumors, which are richer in T cells, are more likely to respond to IT and radiation can potentially convert cold tumors into responsive ones. The role of radiation in converting the tumor to anti-CTLA-4 responsiveness has been demonstrated, where the efficacy of anti-CTLA-4 antibody treatment was seen only after high dose intensity modulated radiation treatment [22, 23•].

In addition to the local effects of radiation, which induces cell death by directly and indirectly inducing DNA damage, there are also three types of nontargeted effects, which depend on the relationship between the irradiated and nonirradiated cell, as well as the proximity to the original site of treatment [24]. These nontargeted effects are believed to arise from the impact of radiation on immune system activation [24]. The radiation-induced bystander effect is a radiobiological effect that is transmitted from irradiated cells to neighboring unirradiated cells, leading to biologic changes in the recipient cells [24, 25]. The radiation-induced abscopal effect is a local radiation-induced systemic effect that extends outside the treated volume and can drive the regression and rejection of nonirradiated, distant tumor lesions [24, 26]. The existence of this type of effect is mainly described in sporadic case reports. Because documented abscopal regressions are rare, its clinical relevance is uncertain with current routinely used radiotherapy regimens [26]. A third type of nontargeted effect, the cohort effect, occurs under heterogeneous irradiation, high-dose-irradiated cells might affect low-dose-irradiated cells, and vice versa; the cohort effect is limited to an area of millimeters within the target [24, 27].

The immune phenotype, or molecular subtyping, of tumors is associated with distinct clinical and biological characteristics that correlate with differential responses to treatment and survival. An example of this is muscle-invasive bladder cancer, where the molecular subtype is classified as luminal, luminal-infiltrated, basal, basal claudin-low, and neuroendocrine-like based on a genomic classifier [28]. Infiltrated tumors, for example, tend to show higher levels of stromal and/or immune cell infiltration [29]. Basal claudin-low tumors although enriched with immune cells, the immune cells have a suppressed antitumor function [29, 30]. The TME (molecular subtype, immune, and stromal signatures) was evaluated for associations with disease-specific survival (DSS) and overall survival (OS) in bladder-sparing trimodality therapy (TMT) bladder cancer patients and in patients treated with neoadjuvant chemotherapy (NAC) and radical cystectomy (RC) [31]. Signatures of T cell activation and interferon gamma signaling were associated with improved DSS in the TMT cohort (hazard ratio 0.30 [0.14–0.65], *p* = 0.002 for T cells), but not in the NAC and RC cohort [31]. Conversely, a stromal signature was associated with worse DSS in the NAC and RC cohort (*p* = 0.006), but not in the TMT cohort [31].

Irradiation has been shown in various preclinical studies to alter tumor immune phenotype by augmenting the presence of MHC I on the tumor cell surface, improving expression of cancer-testis antigens and upregulating the FAS/CD95 complex [26, 32–35]. Studies have also shown that combined ablative dose with low doses of radiation could lead to the reprograming of the immunosuppressive tumor microenvironment (TME) to become more immunogenic and synergistically augment the antitumor response [36]. A preclinical model demonstrated a novel immunological basis for radiation dose fractionation consisting of a single high-dose radiotherapy, followed by post-ablation modulation with four daily low-dose fractions (22 Gy + 0.5 Gy × 4) to reprogram the TME by diminishing immune suppression, enabling infiltration of effector cells and increasing efficacy of tumor control with increased survival [37].

Both conventional fractionation and ablative radiation regimens have limitations in terms of immunosuppressive effects and potential toxicity to surrounding normal organs, respectively. Novel radiation modalities, such as spatially fractionated radiotherapy and ultrahigh-dose rate FLASH irradiation, which venture even further from conventional paradigms have shown promise of increasing the therapeutic index of radiation therapy with the potential of immunomodulation [3].

## Novel Radiation Paradigms

Radiation effectiveness and side effects depend on how the beam is delivered to a patient (i.e., broad, narrow, or micro in size), the absorbed dose delivered (including dose per fraction, dose rate (hypo, hyper, or FLASH)), and even whether the tumor is irradiated uniformly simultaneously (volumetric irradiations), or using a scanning beam (a composition of 1D/2D dose paintings of the tumor), or nonuniformly irradiated, using spatial fractionation where only subvolumes of tissue and tumors are exposed to large radiation doses during a treatment session [17]. Limiting radiation to subvolumes allows for higher radiation doses to be administered, which may otherwise be prohibitive in conventional volumetric radiotherapy due to the resultant toxicities of the surrounding normal organs [17]. Spatially fractionated radiation therapy (SFRT) delivers highly nonuniform dose distributions instead of conventionally practiced homogeneous tumor dose and has shown high rates of clinical response with minimal toxicities in large-volume primary or metastatic tumors [38•].

**Fig. 1:**
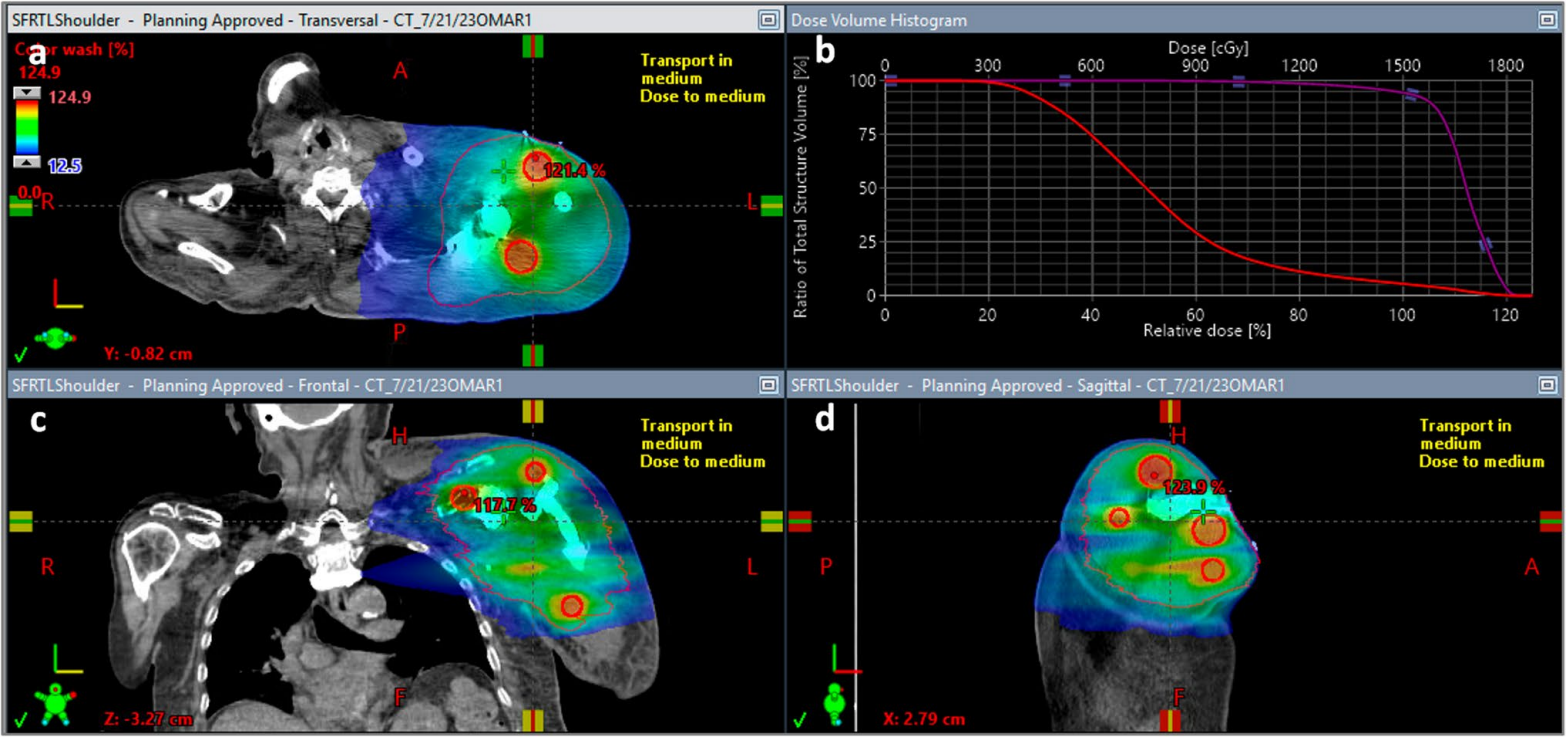
Spatially fractionated radiation therapy with lattice technique. **a**, **c**, **d** Axial, coronal, and sagittal views, respectively, of the SFRT plan with 15 Gy in 1 fraction to the high-dose vertices (red circles). Vertices are 2.5 cm in diameter and placed approximately 3.5–4 cm apart throughout the tumor with sparing of the peripheral 1 cm of the gross tumor volume (GTV). **b** Dose volume histogram representing dose coverage of the combined vertices (purple) and the GTV (red)

The concept of SFRT initially consisted of delivering a high ablative dose to small partial volume(s) within a bulky tumor along with constraining the peripheral doses to the surrounding normal tissue [4]. The main characteristic of SFRT is the peak and valley dose distribution inside the tumor volume [4]. The high ablative dose is delivered to the peaks or vertices while relatively lower doses cover the valley areas between the peaks [4] (Fig. 1). The dependence of radiation effectiveness and side effects on delivery techniques (including spatial and dose fractionation and dose rate) indicates that complex local radiobiological as well as abscopal processes take place as a result of irradiation, including vascular damage/repair and alterations, the response of the immune system, and radiation-induced bystander effects in nonirradiated cells [17]. Alternative methods of radiation delivery might induce unique systemic effects due to the varying damage induced by dose or spatial placement of the beams, and it has been suggested that different dose and fractionation schedules may be a route to more consistent generation of abscopal responses [5••, 6, 7]. It has also been shown that external beam radiation to a target lesion may be sufficient to deplete a majority of circulating naive T cells at critical points of cross-presentation, abrogating the development of effective immunologic antitumor activity and potentially precluding the evolution of abscopal effects [5••, 39]. These findings suggest that SFRT as a component of combination radio-immunotherapy may create interspersed areas of intratumoral immune cell sparing and vascular access with the potential for better immune system activation.

**Fig. 2:**
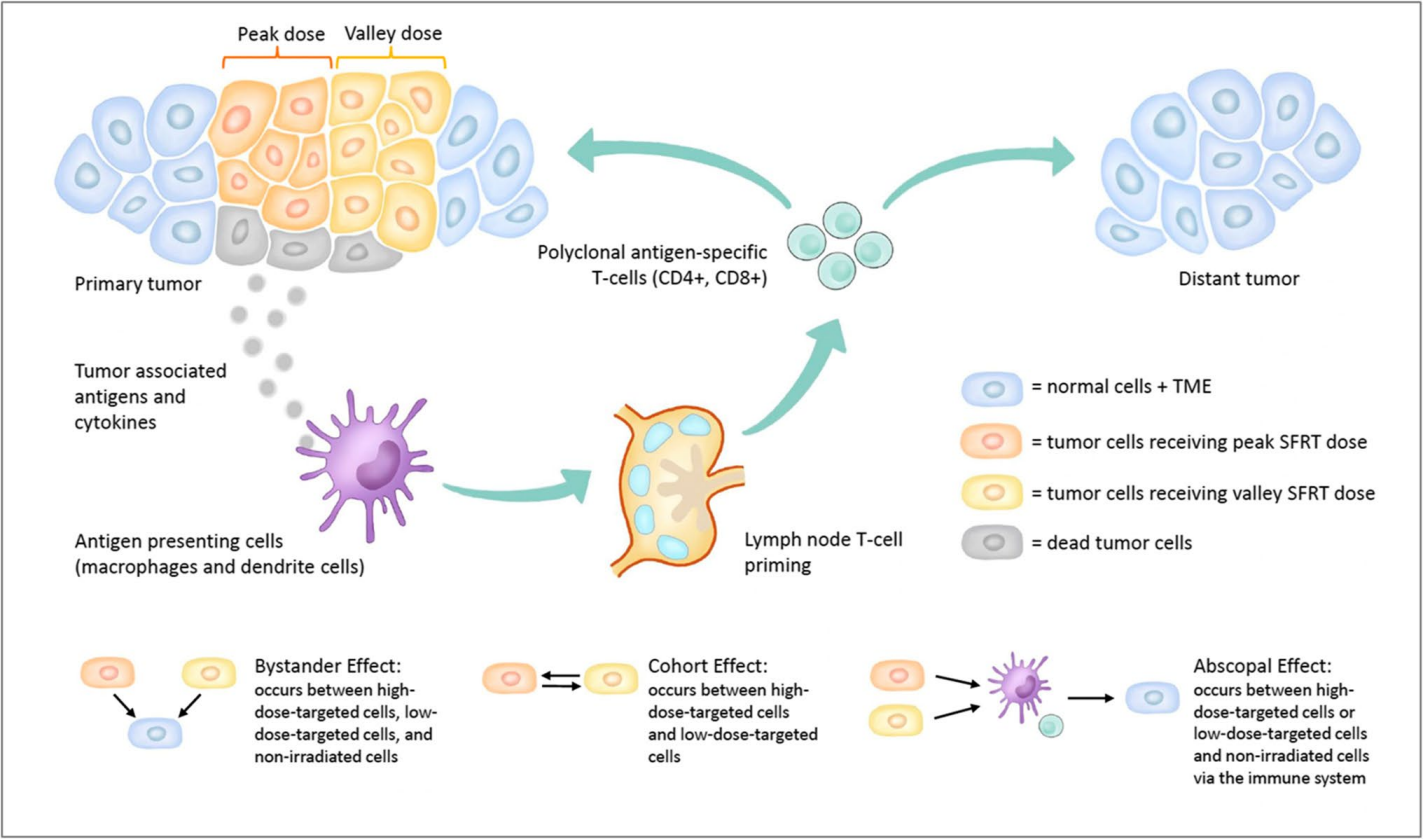
Schematic overview of nontargeted immune effects from SFRT

SFRT can be delivered using GRID [40], lattice (LRT) [41] (Fig. 2), minibeam radiotherapy (MBRT) [42], or microbeam radiotherapy (MRT) techniques [17]. GRID and LRT use centimeter-scale beamlets while MBRT and MRT work with beamlets in the range of hundreds and tens of micrometers, respectively [43]. There are imaging-guided techniques reported that direct the high-dose radiation to specific tumor subvolumes. Oxygen-guided radiation therapy (OGRT) directs high-dose radiation toward hypoxic subvolumes within a tumor using methods such as a simultaneous integrated boost (SIB) [8] or SBRT-PATHY (Stereotactic Body RadioTherapy Partial Tumor irradiation targeting exclusively Hypoxic tumor segments) [44]. Tubin et al. proposed the SBRT-PATHY technique to deliver a high radiation dose to hypoxic areas with a sharp dose fall-off toward the outside of the tumor in order to spare the normoxic portion and the peripheral TME for evoking nontargeted immune radiobiological effects [44]. In this technique, the TME is considered an organ at risk. Ferini et al. [45] used a “metabolism-guided” LRT technique in the LATTICE_01 multicenter study, where the high-dose vertices were placed in the interfaces between the areas of higher ^18^F-FDG uptake (>75% SUV max) and lower ^18^F-FDG uptake [45]. Ferini et al. [46] also describe an “MRI-guided” LRT technique with high-dose vertices targeted to the tumor areas of the highest apparent diffusion coefficient map (ADC)-based signal intensity as a boost. Pollack et al. [36] performed a phase I clinical trial that used LRT to boost 1–3 suspicious multiparametric MRI (mpMRI) gross tumor volumes in the prostate.

## Immunomodulation with SFRT

### Preclinical Immunomodulation with SFRT

Although partial tumor volume is spared direct physical radiation with SFRT, it remains clinically effective by leveraging nontarget effects and TME changes [47]. Most evidence regarding the underlying biological mechanisms of response to SFRT on tumor and normal tissue response has come from animal studies [17]. Observed radiobiological effects from SFRT have been largely grouped into the interrelated areas of vascular, immunological, bystander, and abscopal effects [17]. Focusing on the immunological effects, several preclinical studies using various animal models showed that the highly heterogeneous dose deposition achieved with SFRT is associated with a superior immunological response in tumor tissue [17]. Tumor cell ablation from areas of peak dose is thought to discharge tumor antigen material that enable dendritic priming of T cells. Lymphatic cells, especially CD8+ T cells, are then able to enter tumor tissue via the conserved perfusion of the low-dose areas [17]. Release of interleukins and cytokines or other humoral mediators (e.g., IL-6, IL-8, TGFβ, TNFα, and reactive oxygen and nitrogen species) plays a role in these pathways [8].

Partial tumor volume irradiation using LRT was evaluated using a murine cancer model, where the local and distant effects of whole-tumor irradiation were compared to 20% and 50% volume irradiation delivered to the subcutaneous model of LLC1 lung tumor while the second tumor was not irradiated [48].

The two 10% or one 20% or one 50% volume irradiation using LRT was equally effective as open-field irradiation at eliciting local and distant tumor growth delay coupled with increased immune activation, intratumoral immunogenic death, and antiangiogenic effect. Twenty percent irradiation (in two 10% volumes) showed significant response in both left and right hind leg tumors and further 50% tumor volume irradiation demonstrated effectiveness in the distal unirradiated right tumor. Mice treated with partial tumor volume irradiation induced a robust IFNγ and Th1 response when compared to whole-tumor irradiation and down-modulated TH2 functions. The presence of increased CD3+ cells and TRAIL in partially irradiated tumor volumes correlated well with tumor growth delay. There was a surge in CD3+ T cell infiltration in the unirradiated tumor after 50% tumor volume irradiation. This phenomenon could be exploited in situations where whole tumor irradiation is not possible due to toxicity to critical surrounding normal tissue structures [48].

The importance of CD8+ T cells and adhesion molecules, or ICAMs, in mediating tumor control in nonirradiated bystander areas was shown in a murine model of 67NR breast cancer cells [9, 47]. When 100% of 50% of a tumor volume was irradiated in a single dose of 10 Gy or 20 Gy using X-RAD 225C with a 2 × 2 collimator, there were infiltrations of CD8+ T cells in both irradiated and nonirradiated parts of the tumor at 24 hours after 10-Gy irradiation [9]. The nonirradiated part of the tumor showed a significant increase in endothelial adhesion molecule ICAM that is critical for T cell infiltration [9]. This effect was abrogated if the mice were athymic, treated with antibodies against CD8þ cells, or treated with antibodies against ICAMs, demonstrating the CD8þ T cell dependency of the effect [9]. Immunofluorescence confirmed an influx of primed CD8þ T cells within the nonirradiated part of the tumor [9]. Further investigation revealed that early-infiltrating lymphocytes migrate from the surrounding tissue and the irradiated volume, whereas lymphocytes from lymph nodes maintained long-term antitumor activity [9]. Long-term immunomodulatory effects were noted when reimplantation of 67NR breast cancer cells in these mice failed to produce another tumor [9]. This partial tumor volume irradiation effect, however, was not replicated in a study by Johnson et al. [49], where single fraction of 15-Gy GRID could not initiate an anticancer immune response strong enough to match conventional radiation outcomes. In this preclinical murine study, SFRT GRID was used to treat syngenic transplant tumors induced by intramuscular injection of a soft tissue sarcoma cell line into the gastrocnemius muscle of C57BL/6 mice. Tumor-bearing mice were randomized to four groups: unirradiated, conventional irradiation of entire tumor, GRID therapy, and hemi-irradiation (half-beam block, 50% tumor volume treated). The discrepancy in tumor control compared to the Markovsky et al. [9] study is likely attributable to differences in tumor radiosensitivity and the role of the immune system in radiation response in different tumor models. It should be noted that transplant tumor models have different TMEs than primary tumor models, in which tumors are induced in an organism’s native cells. A primary tumor model would likely provide a more accurate representation of immune responses to cancer and radiation therapy, which would be useful for interpreting preclinical trials to expectations in human treatments [49, 50].

Bystander effects of SFRT are the resulting changes in cells in low-dose areas between the beam tracks [17]. Intracellular gap junctions as well as extracellular soluble mediators are known functional pathways in this mechanism, but the exact nature of molecular signaling is not clear [17]. In preclinical murine models using SFRT, it was found that bystander tumor cells had cell survival rates less than what would be expected with valley doses and had significant overexpression of DNA repair, apoptosis, cell cycle control, heat shock protein, and antioxidant/prooxidant genes [51]. In mice with allogenic Lewis lung carcinoma treated with LRT, there were increases in TNFα and TRAIL after partial irradiation, correlating with tumor growth inhibition [48]. Abscopal effects were also seen in this murine model when irradiating varying tumor volumes of one of the two bilateral tumors [48]. Partial irradiation caused greater tumor growth delay in the distant nonirradiated tumor than did total irradiation, and irradiating two 10% volumes inhibited distant tumor growth more than did one 20% volume [48]. There was induction of cytokines mediating cellular immunity, IL-2 and IFNg, whereas cytokines involved in humoral immunity, IL-4 and IL-10, were downregulated [48]. In addition, there was an increase in the number of infiltrating CD3+ T cells in both the irradiated site and the distant site [47, 48].

The bystander effect in SFRT has been shown to be sensitive to the dose gradient when comparing broad beam irradiation of both tumor and normal tissue with different spatial arrangements of MRT. Survival fractions were determined by clonogenic assay [17, 52]. Cells exposed to a steeper dose gradient, where beams were spaced by 2.5 mm, showed a significantly smaller survival fraction of tumor cells when compared to conventional broad beam irradiation or MRI with beams spaced at 5 mm [52]. Cell toxicity across the low-dose areas is thought to result from bystander signaling transmitted from the high-dose areas where steepness in gradient affects the transmission and distribution of signaling [52].

After MRT in melanoma mouse models, elevations of monocyte-attracting chemokines CCL2 and CCL5 were observed by enzyme-linked immunosorbent assay after MRT and were found to result in substantial inflow of macrophages, natural killer cells, and CD4+ and CD8+ T cells [17, 53]. The MRT peak/valley dose was 408 Gy and 6.2 Gy compared to a conventional broad beam dose of 6.2 Gy [17, 53]. MRT of 9L gliosarcoma tumors in murine models showed significant elevation of immune cell infiltration with CD68 macrophages not seen with the conventional irradiation [17, 54]. The immune activity was in both marginal and central areas of the tumors at 7 days and 14 days post irradiation [17, 54]. Transcriptomic studies give evidence of different genetic pathways of response to MRT versus conventional broad beam irradiation, suggesting a possible biological mechanism behind the greater efficacy observed in tumor control with SFRT [17]. Overexpression of genes after MRT including Cdl9 and the human leukocyte antigen gene complex (HLA) family members is responsible for encoding the major histocompatibility complex (MHC) Class II. Ccl9 is responsible for the CCL9 chemokine known to recruit immune cells to tumor sites and found to have an immunological role in signaling antileukemic responses [10, 17, 55].

### Clinical Immunomodulation with SFRT

SFRT has been shown to support bulky tumor control probability without significantly increased toxicity, as reported by a systematic review of LRT by Iori et al. [56], which included 7 case reports, 1 case series, and 4 clinical studies. When a complete response was not achieved 3–6 months after LRT, a median lesion reduction of approximately ≥ 50% was observed [56]. The systematic review focused on acute toxicity [56].

This review included a case report by Schiff et al. [57] of tumor lysis syndrome (TLS) in a patient with metastatic endometrial clear cell carcinoma treated with LRT on a phase II clinical trial. Treatment consisted of 20 Gy in 5 fractions with an SIB dose of 66.7 Gy in 5 fractions using 7 high-dose spheres. Her creatinine was 2.07 mg/dL on admission. The authors recommend that patients due to receive therapy for malignancy should be risk stratified for TLS. Predictive factors include type and burden of malignancy, anticipated response to cytoreductive therapy, CKD, and pretreatment uric acid and lactate dehydrogenase. Preemptive management may include the monitoring of laboratory values as well as prophylaxis with intravenous fluids and dose-adjusted allopurinol [57]. The review also included an incidence of grade 4 toxicity as reported by Duriseti et al. [58], which consisted of urosepsis after 2 fractions of LRT SBRT to a retroperitoneal tumor with known ureteral involvement as part of the phase I LITE SABR M1 clinical trial. She received medical support and subsequently resumed treatment.

A more recent report describes incidence of grade 3+ toxicity. Owen et al. [59] retrospectively reviewed 126 patients treated with SFRT. Most patients received an SFRT dose of 20 Gy with VMAT placed spheres. Twenty percent of the patients received SFRT alone without subsequent EBRT, 40% of the patients received follow-up palliative SBRT (EQD2 <= 40 Gy10), and 40% of the patients received follow-up definitive EBRT (EDS2 > 40 Gy10). There were 14 grade 3+ toxicity events potentially attributable to any radiation therapy (7.5% cumulative incidence at 6 months, 13% at 12 months). Grade 3+ toxicities included 2 nonhealing wound//skin ulcers, 7 fistulae (bowel/esophageal/bronchopleural), 1 esophagitis requiring hospitalization, 1 grade 3 enteritis and gastrointestinal bleed, 2 radiation pneumonitis, and 1 radionecrosis of the cervix. It was noted that some of the fistulae post radiation were from disease progression following SFRT + EBRT [59].

Most of the reported clinical studies of SFRT focus on clinical outcomes and toxicity, but there are some that also assessed the underlying immune response. Sathishkumar et al. [60] investigated whether circulating cytokines could be measured in patients treated with high-dose GRID radiation and to correlate the finding of these cytokines with clinical response to treatment. TNFα, a cytokine associated with tumor killing, was induced from baseline levels in 32% of patients treated with GRID therapy and was correlated with improved clinical response, whereas TGFβ, a cytokine putatively associated with tumor burden, decreased in 50% with no correlation to response rates [60]. The high (60%) incidence of complete clinical tumor response in patients with TNFα induction can be explained by the possible switching on of signal transduction involving the interaction of TNFα with its cell receptors leading to the activation of the apoptotic pathway [60]. The 23% of patients showing complete clinical response but no TNFα shows that there may also be factors other than the TNFα pathway(s) leading to apoptosis or that TNFα levels were induced over time points not measured in this study [60]. The 50% of the GRID patients showing downregulation of TGF-β1 could be a good index reflecting the reduced tumor burden though no positive correlation to tumor response was observed [60]. However, the downregulation of TGF-β1 may be an additional indicator in our patients having a low risk of developing post-radiotherapy fibrosis [60]. The authors concluded that SFRT producing the combination of enhanced induction of TNFα with its tumor killing and radiation sensitization effect together with the downregulation of TFG-β1 conferring a protective effect on normal tissues may provide the most advantageous therapeutic gain factor in allowing treatment of large and bulky tumors that exceed the potential for control with conventional radiation treatments [60].

It is known that high-dose radiation can increase the immune response against cancer cells when given with ICI, but this dose range has been mainly used for ablative purposes [8]. In the case reported by Jiang et al., high-dose LRT was combined with anti-PD1 immunotherapy in a patient with NSCLC with multiple metastases [41]. One of the metastatic lesions measuring 63.2 cc was treated with high-dose LRT (20 Gy) prescribed to six high-dose vertices combined with anti-PD1 immunotherapy and a complete local response was achieved [41]. With only 6.5% of the GTV receiving a dose of 20 Gy and higher, the effective uniform dose (EUD) of the GTV was calculated to be 1.2 Gy. This tumor was unresponsive to initial anti-PD1 treatment. The other lesions achieved a partial response, including the ones treated with SBRT and LRTs with 10-Gy and 12-Gy vertex doses. This implies that not only a high dose (20 Gy or higher) is essential, but that the spatial fractionation with peak-valley alternation within the tumor volume might also be critical to mediate effective antitumor immune response. This is consistent with other research favoring high dose for effective antitumor T cell priming, and that when combined with low-dose treatment, radiation-induced immune modulation might be augmented. To summarize the postulated mechanism, in high-dose LRT the dose in the vertices is sufficiently high to induce neo-antigen release and initiate the cascade of antigen-presenting cell (APC)-based T cell priming; the dose between the vertices is low enough to preserve internal tumor circulation/perfusion to potentially facilitate the infiltration of APCs and the primed cytotoxic T cells; the highly heterogeneous dose configuration could reprogram the immunosuppressive TME to become more immunogenic; and when synergistically treated by checkpoint inhibitors, the primed T cells could attack tumor cells without being exhausted [41]. Abscopal response of the other tumors with appreciable magnitude was not observed [41].

Bulky tumors are characterized by a heterogeneous oxygen supply that generates hypoxic area, which increases the cancer cell survival fraction from radiation both in vitro and in vivo [61]. Hypoxic clones may develop a metabolic adaption to hypoxia and survive through the hypoxia-inducible factor 1α (HIF-1α) signaling pathway. They may not be actively proliferating but still viable and able to escape from radiation effects [61]. Tubin et al. [44] treated 23 patients with bulky tumor using SBRT-PATHY in order to induce the bystander effect. The hypoxic tumor segment called the bystander tumor volume (BTV) was defined using PET and contrast-enhanced CT, as a hypovascularized-hypometabolic junctional zone between the centrally necrotic and peripheral hypervascularized-hypermetabolic tumor segment. The BTV was irradiated with 1–3 fractions of 10–12 Gy prescribed to 70% isodose-line. The pathologic lymph nodes and metastases were not irradiated in order to assess the abscopal effect. No patient received systemic therapy. At median follow-up of 9.4 months, 87% of patients remained progression-free. The bystander and abscopal response rates were 96% and 52%, respectively. Median shrinkage of partially irradiated bulky tumor was 70%, while for nonirradiated metastases it was 50%. No patient experienced any acute or late toxicity. One patient underwent subsequent surgery and histological evaluation showed massive necrosis in the bulky squamous cell lung cancer with a dense reaction at the border of the necrosis by infiltration of lymphocytes. Approximately 20% of the tumor cells were alive. A separate lung lesion was an 80% necrotic adenocarcinoma, but did not show infiltrating lymphocytes at the borders of the tumor. Three metastatic lymph nodes were completely necrotic [44]. Apoptosis-inducing factor (AIF) was upregulated in the carcinoma cells at all three tumor sites. CD20+ B lymphocytes showed focal aggregates around the bulky tumor, a few were seen within the lymph node metastases, but were absent in the separate lung adenocarcinoma. CD3+ T lymphocytes densely infiltrated the bulky tumor and were prevalent in the lymph node metastases, but were scarce in the adenocarcinoma. These T lymphocytes were predominantly CD8+ cytotoxic lymphocytes, whereas CD4+ T lymphocytes were scarce in the adenocarcinoma and lymph node metastases and were mainly absent in the bulky tumor. CD56+ NK cells were not present at all three tumor sites. S100 protein antibodies predominantly stained macrophages at all three tumor sites. Myeloid-derived suppressor cells were predominantly stained by CD14 and were numerous at the bulky and lymph node tumor sites, but were less intense in adenocarcinoma. CD15+ myeloid-derived suppressor cells were seen in small numbers at all three tumor sites. These results support their preclinical findings that high-dose irradiation of the hypoxic tumor selectively was able to generate stronger nontargeted tumor killing and proliferative block compared with those generated by the normoxic tumor [62]. AIF upregulation in all three tumor sites appears to indicate a significant apoptosis induction, also at unirradiated tumor sites, whereas significant lymphocyte infiltration was only seen at the bulky tumor but the same signs of immune system activation at abscopal site of the lung adenocarcinoma were absent [44]. Since AIF is a mitochondrial protein related to the cytochrome C apoptosis pathway, this suggests an alternative pathway activation and upstream gene(s) for this activation need to be studied further. The observation that a massive necroptosis occurred at an abscopal site, while devoid of signs of immune system activation, supports a hypothesis that an important role is played by abscopal tumor signaling. This is also the first report showing the existence of a crossed radiation-induced abscopal effect, observed after regression of an unirradiated adenocarcinoma following irradiation of a squamous cell carcinoma [44]. Partial-tumor irradiation sparing of the peritumoral immune-environment, and consequent shifting of immune-suppressive to immune-stimulatory effect, may improve radiation-directed tumor cell killing by adding to it a component of immune-mediated killing [63]. A high-single-dose irradiation of hypoxic tumor cells generates a stronger bystander effect and abscopal effect than the normoxic cells, suggesting their higher “immunogenic potential” [63]. Tubin et al. [63] also found interferon gamma, IL-6, TNFα, and TRAIL in abscopal sites and may mediate the systemic antitumor response.

Tubin et al. [63] note that many proinflammatory cytokines are now also considered to be immune-suppressive and are bimodal, which may help explain the dichotomous observations of the immune-stimulatory or immune-suppressive effects of radiation. Cytokines also have short half-lives, low concentrations, and spatiotemporal interactions. The suppressed antitumor response has been shown to dynamically oscillate over approximately 7 days repeatedly in a homeostatic fashion. Tubin et al. [63] hypothesize that radiation-induced cytokine release might skew the immunosuppressive circuitry of radiation-spared PIM to specifically break local tumor tolerance and cascade systemically to deliver bystander and abscopal effects. This cytokine production would have to occur at a specific time to sufficiently extend the normal narrow half-life physiologic restrictions. This temporal window could be as narrow as a few hours every several days [63]. They are investigating the timing of radiation to complement the cytokine immune dynamics in an ongoing prospective trial, but an initial report of eight patients treated with time-synchronized immune-guided SBRT-PATHY showed improved bystander and abscopal responses in those patients treated on the “most favorable day” [64].

## Radioimmunotherapy Strategies Using SFRT

The combination of SFRT and immunotherapy was investigated by Johnsrud et al. [5••], using an immunocompetent mouse model with a triple-negative breast tumor (4T1). A single fraction of 20 Gy was delivered using whole-tumor radiation or GRID alone or in combination with antibodies against immune checkpoints PD1 and CTLA-4. Whole-tumor radiation with ICI significantly restrained tumor growth in the irradiated tumor, but not abscopal tumors, compared to either of these treatments alone. In mice that received GRID, evidence of abscopal immune responses was observed in contralateral tumors with markedly enhanced infiltration of both antigen-presenting cells and activated T cells, which were preceded by increased systemic INFγ production and led to eventual tumor growth delay. PD-L1 was found to be upregulated in abscopal tumors from GRID-treated mice. The intratumoral immune cell composition of abscopal tumors showed significantly increased amounts of both activated CD4+ and CD8+ T cells. The findings illustrate the beginnings of what appears to be a mounting antitumor immune effect that could be instrumental to developing long-term abscopal tumor control. Indications of a protumor control immune cell phenotype began to emerge within days 12–14 post-irradiation [5••]. The was a significant increase in MHCII+ positivity in abscopal tumors, including antigen-presenting cells (APCs) such as dendritic cells, macrophages, and B cells. Conventional dendritic cells (MHCII+/CD11c+) were more abundant in abscopal tumors from GRID-treated mice compared to control or whole-beam irradiation. Though MHCII expression is not entirely exclusive to antigen-presenting cells, together these findings could imply that immunogenic effects of GRID lead to increased antigen presentation, subsequent T cell activation, and general immune response against abscopal tumors. Alternatively, increased infiltration of APCs might represent subsequent events after massive tumor cell death in the primary tumor rather than what would be viewed as a classical initiation of an immune response [5••].

Previous animal studies demonstrated that the generation of abscopal effects requires recruitment of BATF3-dependent dendritic cells to otherwise poorly immunogenic tumors, which are required for cross-priming of tumor-specific T lymphocytes [5••, 65]. The trigger for this response appears to be the accumulation of intracellular dsDNA under the regulation of three-prime repair exonuclease 1 (TREX1) and was only observed after repeated doses (6 and 8 Gy) but not after a single 20-Gy dose, suggesting that a certain dose threshold may be required for successful DC recruitment and T cell activation [5••, 6]. It was also somewhat unexpected that PD-L1 expression, an established biomarker for predicting response to anti-PD1 therapy, was higher than whole-tumor irradiation in abscopal tumors from both GRID and GRID with ICI-treated mice by day 12 [5••]. In agreement with this rise is a published study that established that higher levels of IFNc lead to increased expression of PD-L1 in tumors [5••, 66]. An abscopal, radiotherapy-driven upregulation of PD-L1 is not homologous to native microenvironmental regulation of PD-L1 and therefore may not have the same predictive value for treatment or progression; however, it is possible that treatment of primary tumors with GRID may upregulate PD-L1 at distant sites through the increase of IFNc and render them more susceptible to anti-PD1 therapy and promote a more immune-permissive state [5••].

The combination of MRT with anti-CTLA-4 immune checkpoint inhibitor was shown by Bazyar et al. [67] to increase survival in a murine melanoma model. An immuno-histological analysis showed that MRT recruited cytotoxic lymphocytes (CD8) while suppressing the number of regulatory T cells (Tregs). Using RT-qPCR, it was observed that MRT significantly increased and did not saturate CXCL9 expression, which is a cytokine involved in the attraction of activated T cells. A complete response rate of 50% was observed with MRT combined with anti-CTLA-4 compared to the combination of conventional radiotherapy and anti-CTLA-4. The combination of MRT and anti-CTLA-4 enabled the establishment of long-term antitumor immunity, for which MRT alone failed [67, 68].

Two clinical case reports have been published regarding the combination of SFRT and IT for bulky tumors. Jiang et al. [41] combined high-dose LRT and immune checkpoint inhibitor blockade for a patient with NSCLC with multiple metastases. One of the metastatic lesions measuring 63.2 cc on the posterior chest wall was treated with high-dose LRT combined with anti-PD1 immunotherapy. 6.5% of the GTV received the prescribed vertex dose of 20 Gy, and the valley dose between vertices was approximately 25% of the peak dose. The metastatic mass regressed 77.84% over 1 month after the treatment and had a complete local response (CR) 5 months after the treatment [41]. None of the other lesions receiving palliative treatments achieved CR [41].

Massaccesi et al. [23•] reported on the effects of immune-sparing MR-guided partially ablative irradiation (ISPART) for a patient with bulky peritoneal metastasis (9.2 × 7.5 cm) from renal cell cancer, progressing after sequential therapy with sunitinib and cabozantinib. The tumor was resistant to immunotherapy with nivolumab, given as third-line therapy. To palliate pain and possibly enhance the immune response, the patient was then also treated with ISPART. The target volume (TV) for radiation consisted of the central necrotic core of the mass with no added margins. The peritumoral tissue was considered an organ and was obtained by adding an isotropic 1-cm margin to the gross tumor volume (GTV) and then subtracting the same GTV. A single fraction of 10 Gy was prescribed to cover the TV-ISPART with the 80% prescription isodose line. No acute side effects were reported, except a transient flare-up of pain the day after the last ISPART session. A follow-up CT scan 2 months later showed a 30% reduction in size of the tumor. The authors conclude that ISPART is feasible in bulky renal cell cancer lesions treated with checkpoint inhibitors because of its potential additive or synergistic role. They highlight the importance of avoiding radiation to doses higher than 2 Gy to the peritumoral tissue, which is the zone containing lymphocytes.

## Conclusion

SFRT is of increasing interest in clinical, experimental, and translational radiation oncology [38], and future research is needed to understand the underlying immune effects of SFRT including nontargeted effects. Important goals include optimizing radiation doses and fractionation strategies to achieve immunomodulation to enhance the effects of IT. Questions remain as to the optimal spatial distribution of the high-dose vertices (geometric, hypoxia-targeted, or other image-guided approaches) and optimal methods to spare the TME. Whether to deliver SFRT in a single fraction or fractionated approach, as a boost or alone, and/or combined with systemic therapies needs further evaluation. The optimal dose including whether there is a threshold dose required for DC recruitment and T cell activation is also unknown [5••]. Particularly in combination with ICIs, the question is whether SFRT can prime the immune system to augment bystander, abscopal, and cohort responses [3] or even alter the molecular subtype or immune phenotype of the primary tumor by increasing levels of immune cells within the tumor. Whether SFRT increases the access of the immune system into the TME or simply does not induce as strong a suppressive immune response compared to whole-tumor irradiation remains to be determined [5••]. The timing of partial volume radiation is also being further investigated to synchronize with immune system oscillation [64]. Most SFRT treatment has focused on bulky tumors, but there may be a role for use in the neoadjuvant setting to possibly improve complete responses prior to surgery or to augment waning effects of IT. Combining SFRT with ultrahigh-dose delivery is also an area for further research [43]. Correlation with peripheral blood and tissue immune activation assessments, molecular subtyping analyses, and “omics” such as radiomics [69] is essential for biomarker discovery.

In summary, radiation to tumor subvolumes has potential for immune system activation and possible other pathway activation for abscopal responses, and prospective clinical trials are needed to address the underlying mechanisms of SFRT in order to improve the therapeutic ratio of radiation and to increase the efficacy of IT.
